# Experimental and Numerical Analysis of Multiple Low-Velocity Impact Damages in a Glass Fibered Composite Structure

**DOI:** 10.3390/ma14237268

**Published:** 2021-11-28

**Authors:** Kaleeswaran Balasubramaniam, Dominika Ziaja, Michał Jurek, Piotr Fiborek, Paweł Malinowski

**Affiliations:** 1 Department of Mechanics of Intelligent Structures, Institute of Fluid Flow Machinery, Polish Academy of Sciences, Fiszera 14, 80-231 Gdansk, Poland; kaleeswaranb@imp.gda.pl (K.B.); pfiborek@imp.gda.pl (P.F.); pmalinowski@imp.gda.pl (P.M.); 2 Department of Structural Mechanics, Rzeszow University of Technology, Al Powstańców Warszawy 12, 35-959 Rzeszow, Poland; dziaja@prz.edu.pl

**Keywords:** glass fibered reinforced polymer, impact damage, guided waves, laser vibrometry, spectral elemental method, digital image correlation

## Abstract

Glass fiber-reinforced polymer structures (GFRPS) are widely used in civil and mechanical fields due to their light weight and corrosion resistance. However, these structures are prone to damage with very-low-energy impacts. The reliability of such structures is of prime importance before their installation and usage. This study aimed to identify, visualize, localize, and verify multiple barely visible impact damage (BVID) in a GFRPS using a combination of guided waves (GW)-based online structural health monitoring (SHM) and thermal strain-based nondestructive testing (NDT) approaches. Global NDT techniques like the use of a laser Doppler vibrometer (LDV) and digital image correlation (DIC) were used in the experimental analysis. The effectiveness of the experimental LDV-GW process was also checked numerically with the spectral element method (SEM). A threshold-based baseline free SHM approach to effectively localize the damages was proposed along with quick DIC verification of composite structure with thermal loading based on short-pulse heating as an excitation source. This study analyzed combined experimental- and numerical-based SHM-NDT methods in characterizing the multiple BVIDs located in a GFRPS.

## 1. Introduction

Composites are light weight, have high strength, and are used in many automotive, civil, aerospace, etc. applications. The GFRPS laminates have greater strength along the direction of fiber [1] GFPRS are widely used in retrofitting structures in construction industries, and, nowadays, these glass fibers are combined to prepare reinforcement bars for structural strengthening [2]. They are highly preferred due to their high tensile strength, low density, and corrosion resistance [3]. Even though several advantages exist, they also have disadvantages like delamination defects, impact cracks [4], internal fiber matrix damage [5], etc. If such damage fails to be noticed, it could lead to severe structural disintegration [6]. BVID and impact cracks in the GFRPS are of most common that occurs even due to a low-energy impact force and are mostly not visible for internal visual identification.

The engineering industry offers many methods for analyzing the defects in the structures. The NDT analysis of glass fibers using a combination of X-ray tomography, ultrasonic imaging, and optical proliferometry was proposed by Miqoi et al. [7] and on carbon fibers using digital image correlation (DIC) and scanning electron microscopy by *Sarasini* et al. [8], in addition to experimental compression after impact tests by Talreja et al. [9]. SHM finds its way in detecting such impact damage by performing continuous health monitoring of structures 1. SHM reduces the laborious human hours of checking the whole structure for damage with the help of fewer piezoelectric lead zirconate titanate (PZT) to analyze the structure. These PZTs send and receive the signals and thus help in online monitoring.

GW are of recent trends in SHM applications due to their higher area coverage in a short interval of time. The GW analyzes the damage based on differences in the attenuation parameters [4] The GWs are classified into symmetric (S) and antisymmetric (A) waves based on their velocity, varying propagation patterns, etc. The slower GW mode is the A0 mode in lower frequencies, and the faster one is in the S0 mode [10,11]. The electrical input applied to the PZT results in the excitation of GW signals, which could be used for online monitoring of structures [1].

The impact damage on glass fibers was analyzed using wavelet packet transform [12]. Impact damage sensing based on fast Fourier transform peak analysis, nonlinear sideband peak variations, and time-of-flight analysis were performed on GFRP plates by Alnuaimi et al. [13]. The impact effects in the GFRP structure were studied using GW amplitude variation due to defect size, fiber breakage [14], and phase difference [15] with a circular PZT network. Zhang et al. used differences in mode conversion behaviour to analyze the glass fibered structure at different stages of impact [16]. LDV-based root mean square [17] and radially weighted root mean square (RRMS) formulations [18] were used by many in analyzing the entire area of composite structures. Numerical-based SEM codes were also used in studying impact damage [19]. A combination of experimental and numerical methods using the SEM was used in detecting the impacts in composite structures [20]. A comparison of BVID identification was performed [21] using the signal-to-noise ratio damage index method and the probabilistic damage algorithm.

The DIC method uses the analysis of changes in the random speckles pattern coated on the surface of the examined object, and the observation of displacements or strain field variation is possible. In many cases, these changes inform about damage appearance before the defects are visible [22]. An additional advantage of DIC is the possibility of simultaneous measurement of many points of the observed area; for this reason, this method was chosen as a verification technique in this study [23].

In many DIC applications, the specimens were tested in universal testing machines, where the test finishes with specimen destruction [24,25]. The main problem of applying DIC as a nondestructive method is the determination of the way the sample is loading such that the observed displacement/strain field is sufficiently large concerning the accuracy of the measurement and, at the same time, fully reversible. The short heating process was selected and proposed as an excitation in the presented approach to reduce the verification time. The effectiveness of the DIC technique for full-field high-temperature thermal deformation measurement has been demonstrated, e.g., in [26] where the authors examined the thermal properties of austin stainless steel. The thermal effects in composite specimens using DIC were examined by Foti et al. [22], but the authors examined the high-temperature fatigue. The thermal buckling of the circular composite plate was analyzed based on DIC measurement by Jin et al. [27] and Zhu et al. [28] who used DIC to observe the cooling process of turbine vane thermal barrier coatings after thermal shock. At the same time, Rajaram et al. [29] showed the utility of DIC to surface damage detection of aluminum specimens based on the coefficient of thermal expansion.

Most research works focus on the localization of single-impact damage during and after the impact [5,8,10,12,15]. There are fewer research works on analyzing and localizing the multiple impacts and on verification of the deformed areas after impact. In our research work, we focused not only on identification, visualization, and localization of the multiple linearly located impact damages but also studies were carried out to observe changes (verification) in the impact-deformed zones by applying a modified [23] quick thermal strain field technique.

A numerical method based on SEM code was developed and compared with the experimental LDV signals to analyze the damage. Since all three damages existed before, the numerical simulation was utilized to study each damage scenario separately. The RRMS method was applied to the signal data obtained from both LDV and SEM. The SHM-based baseline free elliptical threshold method (ETM) was proposed to show the effective online localization of the damages. Thus the proposed SHM-GW technique can be used as the first step of the quick analysis to locate the damages followed by NDT-DIC verification based on SHM results. The SHM combined with the NDT method can drastically reduce the time to analyze larger and complex structures.

## 2. Methodology, Material, and Approaches

### 2.1. Methodological Flow Chart

**Figure 1:**
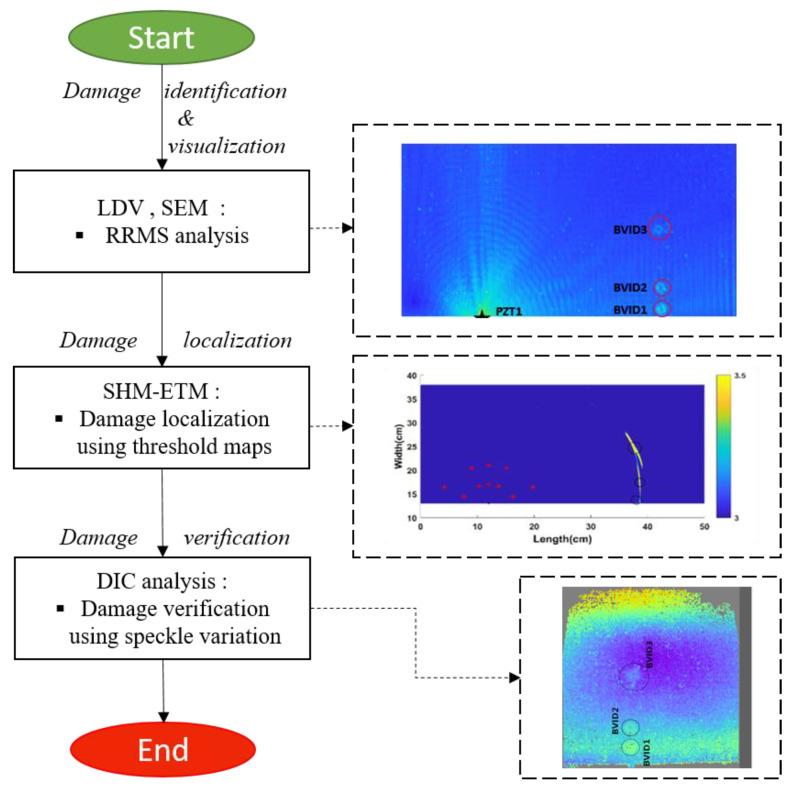
The methodological flow chart in damage analysis.

The flowchart (Figure 1) briefly explains the methodological process involved in analyzing the GFRPS using combined NDT and SHM methods. The process involves damage-identification, visualization, localization, and verification.

### 2.2. GFRP Material Studied

**Figure 2:**
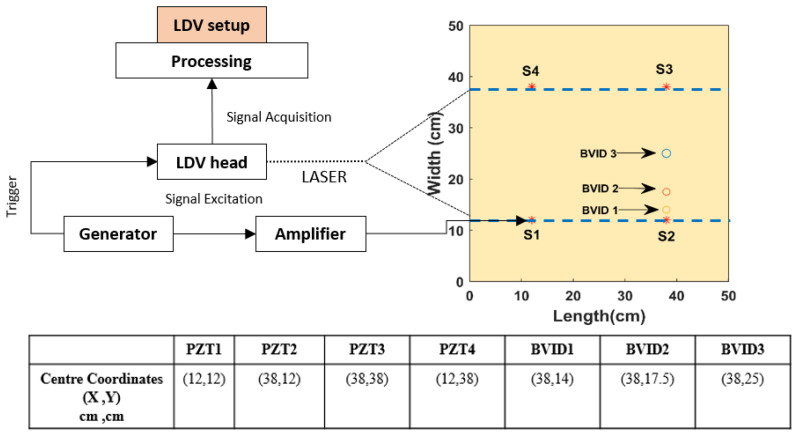
Schematic LDV experimental setup used with PZT and BVID coordinates.

The 0.2 cm thick GFRPS has 12 layups stacked in [0/90]_3s_ format, which are bonded together by HEXCEL-212Na adhesive. The material is procured from the G.ANGELONI group [30] (Venice, Italy). These materials are largely used in the mechanical and construction industries. The GFRPS is of dimensions 50 × 50 cm^2^ in length and breadth. Multiple BVIDs of Ø 0.9 cm (BVID1) and Ø 1.2 cm (BVID2 and BVID3) are made with an impact force of 20, 25, and 30J. This is done by dropping a steel ball attached to a steel beam from varying heights guided via a pipe. All the 4 PZTs (Ø 1 cm and 0.1 cm thin) are made from SONOX material P502 [31] and are attached to the GFRPS using cyanoacrylate glue. The PZTs (S1–S4) and BVIDs location coordinates are presented in Figure 2.

### 2.3. LDV Setup

A single head (1D) LDV was chosen, which analyzed the structures using out-of-plane wavefield components. Polytec PSV 400 was the LDV type used. LDV helps to visualize the GW propagation and also to acquire the GW full-wave fields. The GW propagation was done by exciting the PZT. The retro-reflective film was applied only to an area of 26 cm × 50 cm (blue dotted lines), as shown in Figure 2, to reduce the LDV calculation time. The LDV setup consisted of the signal generator, the power amplifier, and the oscilloscope. A 5-cycle sinusoidal tone burst generated with a Hanning window was applied to the PZT. Frequencies of 50 kHz, 100 kHz, and 200 kHz were used in this analysis. The LDV measurement at each grid point was time-averaged 5 times with an overall sampling period of 1024. A 16 V_pp_ voltage with a gain of 20 was applied to PZT via the generator. All four symmetrical positioned PZTs were excited via this process.

### 2.4. SEM Numerical Approach

**Figure 3:**
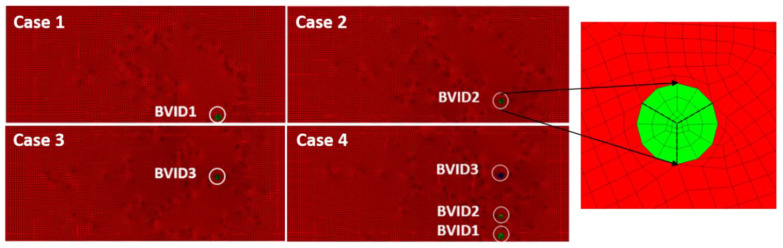
SEM_GFRP model cases studied.

The simulation was conducted with the algorithm developed in Matlab software (version 2020a) based on the implementation found in [32]. The GFRP structure was modelled according to the Mindlin–Reissner first-order shear deformation theory [33], and the laminate theory [34] was used to determine the effective engineering constants shown in Table 1. The details of the determination of the effective constants are given in Appendix A. The model of PZT transducers was omitted in the analysis to simplify calculations. Instead, the out-of-plane forces were applied to the nodes in the place of the actuator. The damage was modulated in two ways. First, Young’s modulus of the structure was reduced by 50% in the circular of the damaged area. Second, three cracks were introduced using node separation [35] at the location marked by the dashed line in Figure 3. The LDV experimental setup values, as mentioned earlier, were used in studying the numerical GFRP models.

The four model cases (Figure 3) were made numerically as the structure obtained already had three BVIDs in the initial state itself. Thus, SEM models added an advantage to visualize how the GW propagation would be with the individual BVIDs.

### 2.5. DIC Setup

**Figure 4:**
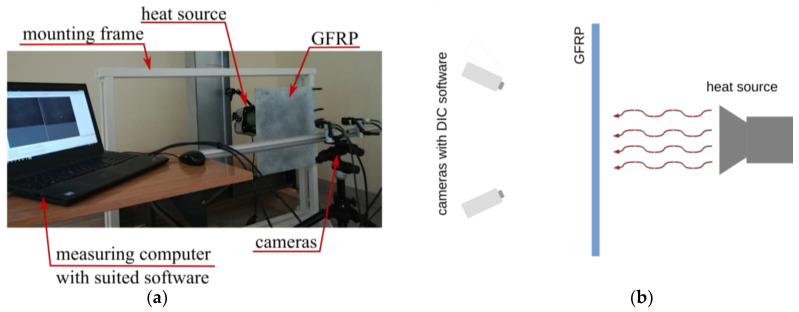
(**a**) DIC measuring stand; (**b**) scheme of the experimental setup.

As a verification procedure, 3D DIC was proposed; so, the measuring setup consisted of two USB3.0 Baumer 12.3 Mpx cameras (Baumer GmbH, Radeberg, Germany) with VS-1220HV lenses, combined in a Q400 system by DANTEC DYNAMICS GmbH. The strain field changes resulted from heating the GFRP-plate using a halogen lamp HEDLER HF 65 (HEDLER GmbH, Hesse, Germany); the exposition time was 2 min. The size of the observed area was a compromise between the highest possible accuracy and the widest observed area. The area observed by each camera was about 340 mm in width and 249 mm high, which considering the camera resolution of 4096 × 3000 px gave the spatial resolution equal to 83 μm/px. The calibration was made using the Al-15-BMB_9×9 calibration target. During the evaluation of pictures, the facet size was established to 17 pixels. In the presented examination, the mean accuracy of the displacement measurement was: in the horizontal direction, 0.00006 mm; in the vertical direction, 0.00007 mm; and, perpendicular to the observed plane, 0.002 mm. The whole specimen was not observed. The measuring stand is shown in Figure 4a, and the scheme of the experiment is shown in Figure 4b, top view.

## 3. Damage Analysis Methods

The damage identification and visualization were done using RRMS for both LDV and SEM data (Section 3.1), localized by the developed SHM-based ETM method (Section 3.2) and verified by the proposed DIC thermal speckle-based variation method (Section 3.3).

### 3.1. RRMS-Based Damage Analysis

The visualization method using RRMS [18] for four PZTs excitations were analyzed. The RMS provides the energy distribution of the signals. However, if more energy gets accumulated in a certain region, it shows a higher value of RMS. It results in blurring of the damage zone sometimes. The RRMS function Equation (1) was added to counteract such cases, which enhanced the results obtained.

(1) $$RRMS\left(x,y\right)=\sqrt{\frac{\sum_{i=1}^{N}{\left(k_{i}\left(x,y,t\right)\right)}^{2}}{N}}\times {\left[RWF\right]}^{a}$$

where $k_{i}$–*i*th sample amplitude, and *N*—the number of registered time samples.

*RWF* is the radially weighted factored value for each scanning point, and “*a*” is the power of the weight factor. In this function, the excitation points were the origin point (*x*_0_, *y*_0_), and the radial distance was measured for all the points (*x_i_*, *y_in_*), as shown in Equation (2).

(2) $${\left(RWF\right)}_{i}=\sqrt{{\left(x_{0}-x_{i}\right)}^{2}+{\left(y_{0}-y_{i}\right)}^{2}}$$

With the help of the obtained Equation (2), a combination of LDV and SEM models were analyzed as mentioned in Table 2.

### 3.2. Threshold-Based Elliptical Method in Damage Analysis

The localization of the damages was done using the SHM-based strategy using the ETM. It works on the methodology of convergence and interaction of ellipses. The method requires the time of arrival (TOA) of the wave peaks in the damage calculation [36,37]. The time of arrival (theoretical) (TOA^T^) was obtained between GW paths to the arbitrary grid points, the sensor, and the actuator. After applying the Hilbert transformation (HT) envelope to the signals, amplitude peaks were obtained. The obtained TOA^T^ was then tried to match up with the HT peak values (TOA^E^).

**Figure 5:**
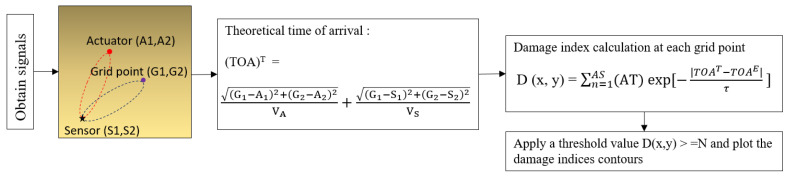
Damage analysis based on ETM.

A higher value in the mesh grid was obtained when the TOA^T^ matched with an HT value. A fine mesh of 0.1 mm spacing was used in the calculation. Since most reflections occurred at the damage spot or near the damage spot, that region was highlighted in the grid. A threshold value (90%) was applied to the algorithm to separate the higher values in the grid during the run. The threshold value was selected based on the experience and the references from the threshold-based probabilistic study [38]. The damage localization approach is explained in a flowchart, as shown in Figure 5.

(G_1,_ G_2_) are arbitrary grid point coordinates; (A_1_, A_2,_ S_1,_ S_2_) are actuator and sensor coordinates; V_A_, V_S_ represents the group velocities obtained from the actuator to damage and the sensor to damage, respectively; A^T^ represents the amplification factor; TOA^E^ represents the time of arrival obtained from the experiment; D (x, y) represents the damage index; AS represents the total number of actuator sensor paths studied; τ is the decay factor value of 5 μs, and N represents threshold value used in the calculation.

### 3.3. Thermal Speckle-Based Verification Method

**Figure 6:**
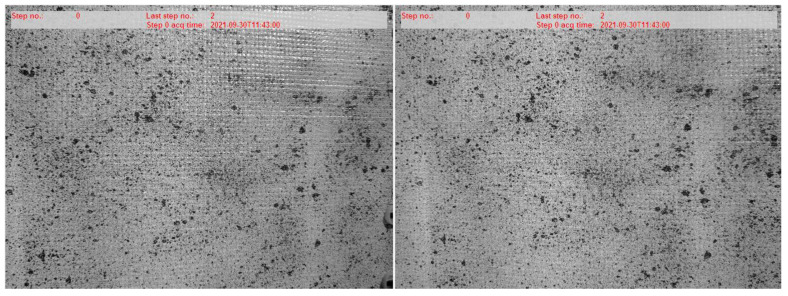
The exemplary view, which was registered by camera no. 1 (**left**) and camera no. 2 (**right**). The defects were invisible.

The examined GFRPS was coated by the typical DIC speckle pattern (registered by two cameras as shown in Figure 6), so the damages were invisible. The specimen was observed under the heating process realized by the light source. Since the damage location was initially localized with SHM, the lamps were focused in the localized area. The reference picture was made at stable room temperature, and then the whole heating took two minutes. During this time, each camera registered two new photos every one second (sampling frequency equal to 2 Hz). For the analysis of picture sequences, the commercial ISTRA 4D software (version 4.6) was used.

## 4. Discussion on the Results Obtained

### 4.1. RRMS Models-Visualization of Damages

**Figure 7:**
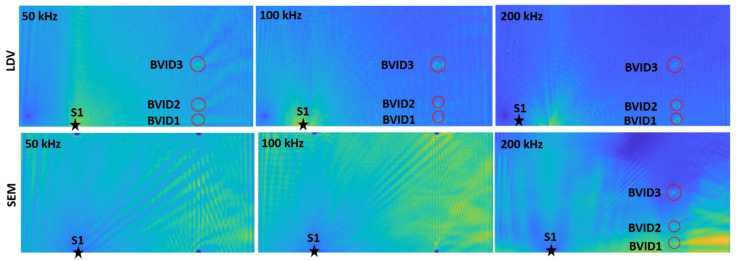
Cases 4 and 5: LDV_RRMS and SEM_RRMS plots for S1 excitation.

**Figure 8:**
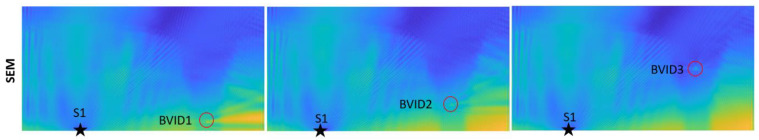
Cases 1, 2, and 3: SEM_RRMS plots for 200 kHz excitation.

RRMS studies were carried out with the LDV and SEM data to visualize the damage. A constant power/weight factor value of *a* = *0.5* was used for all the calculations. Table 2’s case results are shown in Figure 7 and Figure 8. Initially, cases 4 and 5 (Figure 7) were considered for the visualization study as they represent the real damage scenarios.

After analyzing all the frequencies, it was found that both LDV_RRMS and SEM_RRMS 200 kHz plots identified all the BVIDs more prominently with rounded red circles (Figure 7). Table 2’s other cases (1–3) were also checked only with 200 kHz. Thus, only 200 kHz was chosen as the identified frequency for other RRMS and SHM results (Section 4.2). The SEM_RRMS plots, as shown in Figure 8, identified the inner numerically made BVIDs (1, 2, and 3) separately and proved that the methodology can be used for single damages.

**Figure 9:**
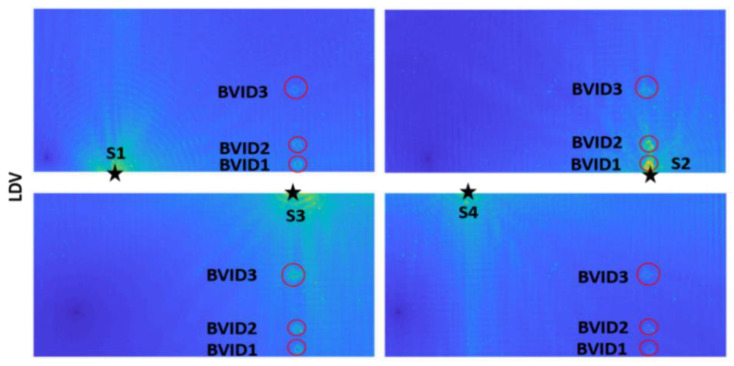

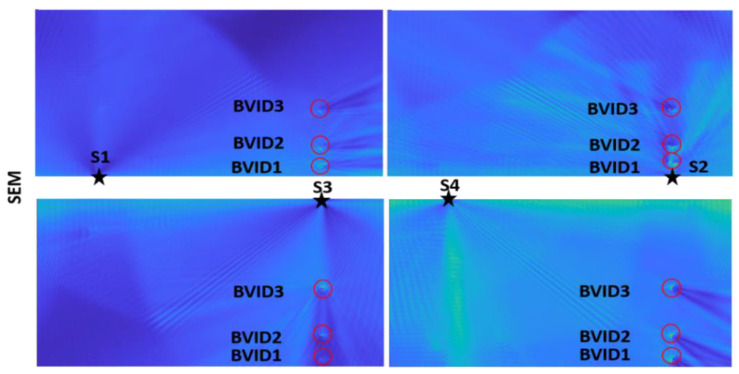
LDV_RRMS and SEM_RRMS plots for 200 kHz excitation of all S1–S4.

After checking the results from S1, similar BVID identification and visualization checks were done for other symmetrically located sensors, as shown in Figure 9, using the RRMS-based weightage formulation. All sensors identified the damages clearly both from the experimental and the numerical data.

### 4.2. ETM-Localization of Damages

**Figure 10:**
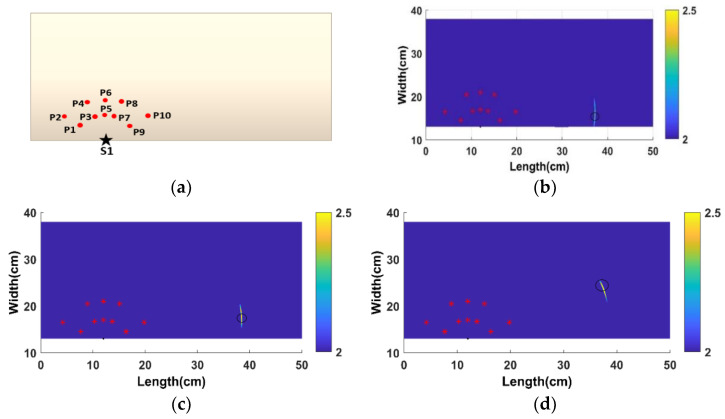

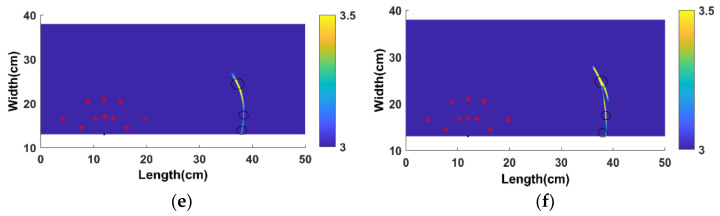
(**a**) Distribution of scanning nodal points taken; ETM for (**b**) case 1, (**c**) case 2, (**d**) case 3, (**e**) case 4, and (**f**) case 5.

The amplification and threshold factor-added elliptical-based algorithm was applied to Table 2 in damage localization. The damage points were identified based on elliptical intersections. Equidistant nodal points (P1–P10) were taken from the LDV area scan results with excitation from S1 (Figure 10a) for the process. Since S2 and S3 are in a straight line to the detected damages, they were not considered for taking the SHM study. After analyzing S1 and S4, the S1 RRMS maps detected the damage better than its symmetrical counterpart S4. A frequency range of 200 kHz was considered for this study, as mentioned earlier. The full wavefield data of the cases showed that the A0 GW mode identified the damage cases clearly, which was chosen to study further. The A0 mode velocity values obtained for the LDV by scanning points at 90° and 0° were 2231 m/s, 1768 m/s, 2135 m/s, and 1677 m/s for the SEM calculations. The velocity profile was obtained by fitting the velocity values to an elliptical function and determining the 360° velocity profile.

The damage localization plots are shown in Figure 10b–f. The maps were plotted for all five cases (4 SEM and 1 LDV), and higher-energy regions were isolated with threshold values. The region shows that higher energy was nearer to the created BVIDs (circled in black). The error estimation is shown in Table 3 and Table 4. The values obtained were closer to the BVID regions and are in an error marginal range of >1 cm difference maximum.

### 4.3. Thermal Speckle-Based Verification of Damages

**Figure 11:**
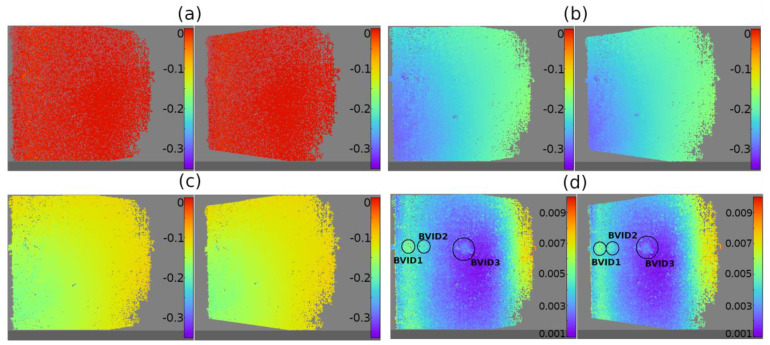
Horizontal displacement of points obtained by DIC measurements: (**a**) the first step (0.5 s of heating), (**b**) the 120 steps (after one minute of heating), (**c**) the 240 steps (after two minutes of heating), and (**d**) the estimated standard deviation of the displacement in the horizontal direction (mm). In (**a**–**c**) the color was kept: from 0.35 mm (violet) to 0 mm (red).

The collected data were carefully analyzed, considering the displacements in three directions and strain fields. In Figure 11a–c, the horizontal displacement of points in the first, 120th, and 240th steps (after two minutes of heating) were shown, respectively. In the figures, the horizontal displacement field was quasi even in each step and corresponds to the boundary conditions (clamping on the right side). In such a short period of heating, there were no visible differences between point displacements in damaged and undamaged regions.

However, the estimated standard deviation of the displacement in the horizontal direction for every data point (shown in Figure 11d) allows to differentiate the failure areas (damages); the standard deviation for damages was greater than for healthy regions in the neighbourhood. Similar observations were made for the other directions.

## 5. Conclusions

In this study, a combined SHM-GW and NDT-DIC method was proposed in studying BVIDs presented in the GFRPS. A frequency range of 200 kHz was analyzed for the GW-based studies. SEM numerical models were compared with the experimental data.

▪ SEM helped to model BVID separately and together (experimental data) to validate the SHM methodology.
▪ The RRMS study provided results about the damage locations even near the higher-energy zones.
▪ S1 provided good analyses of the results, and scanning points were taken to analyze the damage zones.
▪ The threshold-based proposed ETM algorithm predicted the location of the damages.
▪ The error range was less than 1%, as shown in the difference (cm) of Table 4 in all the analyzed cases.
▪ DIC-based diagrams of variation in displacements differentiated the damage region and thus verified it.
▪ Combined online monitoring and verification of the structure was proposed.

Therefore, it is promising to use the proposed SHM-GW and DIC-NDT application in monitoring mechanical, civil structures to verify structural integrity. Future research studies involve implementing similar threshold-based localization and verification approaches in analyzing different structures for certification and reliability purposes.

## Figures and Tables

**Table 1:** Engineering constants for GFRP.

Material	E11	E22, E33	G12	G23	ν12	ν23	ρ	V
	GPa	GPa	GPa	GPa	-	-	kg/m^3^	%
Glass fiber	75.0	75.0	30.70	30.70	0.22	0.22	2600	50
Epoxy	3.40	3.40	1.30	1.30	0.35	0.35	1250	50
GFRP	39.20	7.75	3.41	2.82	0.29	0.37	1925	-

Where E represents the Young’s modulus, G represents the shear modulus, ν represents the Poisson’s ratio, ρ is the density, and V is the volume fraction.

**Table 2:** RRMS cases studied.

Case	1	2	3	4	5
Type	SEM	SEM	SEM	SEM	LDV
Model	BVID 1	BVID 2	BVID 3	BVID 1,2,3	BVID 1,2,3

**Table 3:** Error estimation for cases 1, 2, and 3 (center coordinates).

SEM Cases	Case 1	Case 2	Case 3
Actual location (cm)	(38,14)	(38,17.5)	(38,24)
Estimated location (cm)	(38.3,13.4)	(38.5,17.4)	(37.4,24.1)
Difference (cm)	0.67	0.50	0.60

**Table 4:** Error estimation for cases 4 and 5 (center coordinates).

SEM and LDV Cases	Case 4			Case 5		
	BVID1	BVID2	BVID3	BVID1	BVID2	BVID3
Actual location (cm)	(38,14)	(38,17.5)	(38,24)	(38,14)	(38,17.5)	(38,24)
Estimated location (cm)	(38.7,13)	(38.6,17)	(37.7,24)	(38.3,13.9)	(38.3,17.3)	(37.2,24)
Difference (cm)	0.74	0.60	0.76	0.32	0.34	0.89

## Data Availability

The data presented in this study are available on request from the first, corresponding authors.

## Appendix A

The elastic constants of the unidirectional fibre composite in the form of the Hahn functional are gathered in Table A1.

**Table A1:** Elastic constants.

Elastic Constant	P	P_f_	P_m_	η
E_11_	E_11_	E_11f_	E_11m_	1
ν_12_	ν_12_	ν_12f_	ν_m_	1
G_12_	1/G_12_	1/G_12f_	1/G_m_	η_6_
G_23_	1/G_23_	1/G_23f_	1/G_m_	η_4_
K_T_	1/K_T_	1/K_f_	1/K_m_	η_K_

The Hahn function is given in the form:

(A1) $$P=\frac{\left(P_{f}V_{f}+\eta P_{m}V_{m}\right)}{\left(V_{f}+\eta V_{m}\right)}$$

where $V_{f}$, $V_{m}$ are the volume fraction of the fibers and matrix, respectively. The bulk modulus (K_T_) for fibers and the matrix is equal to $K_{f}=E_{f}/2\left(1-\upsilon_{f}\right)$, $K_{m}=E_{m}/2\left(1-\upsilon_{m}\right)$, respectively, and the expressions for η are given as follow:

$$\begin{array}{c} \eta_{6}=\frac{1+G_{m}/G_{12f}}{2}\\ \eta_{4}=\frac{3-4\upsilon_{m}+G_{m}/G_{23f}}{4\left(1-\upsilon_{m}\right)},\\ \eta_{K}=\frac{1+G_{m}/K_{f}}{2\left(1-\upsilon_{m}\right)}. \end{array}$$

The transverse moduli and $\upsilon_{23}$ of the composite are given:

$$\begin{array}{c} E_{22}=E_{33}=\frac{4K_{T}G_{23}}{K_{T}+mG_{23}},\\ \upsilon_{23}=\upsilon_{12f}V_{f}+\upsilon_{m}\left(1-V_{f}\right)\left[\frac{1+\upsilon_{m}-\upsilon_{12}E_{m}/E_{11}}{1-\upsilon_{m}^{2}-\upsilon_{m}\upsilon_{12}E_{m}/E_{11}}\right] \end{array}$$

where $m=1+\frac{4K_{T}\upsilon_{12}^{2}}{E_{11}}$.

According to the rules, the mixture mass density of the composite is equal $\rho =\rho_{f}V_{f}+\rho_{m}V_{m}$.
